# Development and Validation of a Simple LC-MS Method for the Quantification of Oxytocin in Dog Saliva

**DOI:** 10.3390/molecules24173079

**Published:** 2019-08-24

**Authors:** Lei Wang, Dakota W. Marti, Rachel E. Anderson

**Affiliations:** Nestlé Purina Research, Nestlé Purina PetCare, Saint Joseph, MO 64503, USA

**Keywords:** dog, mass spectrometry, oxytocin, saliva

## Abstract

Oxytocin (OT) is a mammalian neuropeptide with various functions in regulating birth, lactation, parenting, and social recognition. The study of OT became of increasing interest for the petcare industry due to its role in animal behavior and socialization. In the present study, a simple, sensitive, and accurate liquid chromatography-mass spectrometry (LC-MS) method for quantifying OT in dog saliva was developed and validated. OT and its deuterated internal standard (OT-d_5_) were detected with multiple reaction monitoring (MRM) in a positive ion mode using an AB Sciex 6500+ QTRAP mass spectrometer. Chromatographic separation was achieved by using an ACE Excel C18 column and a gradient elution at a flow rate of 0.5 mL/min over a 5 min run. The mobile phases consisted of 0.1% (*v*/*v*) acetic acid in water and 0.1% (*v*/*v*) acetic acid in acetonitrile. After development and optimization, the performance of the method was validated to prove its reliability. Calibration curves were linear over the range of 50–20,000 pg/mL and recovery of OT was above 87.8%. The validated method was successfully applied to evaluate OT concentrations in multiple batches of dog saliva samples.

## 1. Introduction

Oxytocin (OT) is a mammalian neuropeptide containing nine amino acids and has various regulatory functions in multiple conditions such as birth, lactation, parenting, social recognition, and relationship bonding [1,2,3,4]. Besides these functions of endogenous OT, previous studies showed that social affiliations were promoted after intranasal administration of OT in humans [5,6]. Recently, OT drew increasing attention in the petcare industry because of its potential role in animal welfare, such as indicating happiness, as well as affecting affiliative and aggressive behavior [7,8]. Similar to findings in human studies, dogs treated with OT were shown to increase affiliative behavior toward humans and other dogs, and enhanced use of human social cues in object choice tasks [9,10]. 

Due to the simplicity of its collection procedure, saliva has become a promising alternative to other types of samples such as blood, urine, and feces in the field of diagnosis. The analysis of saliva has been explored as a simple and cost efficient way for evaluating homeostasis and pathologic conditions of the body [11]. Compared to other samples, saliva has many advantages as a diagnostic specimen, including easy collection, noninvasive sampling, and low biohazard risk. Conversely, disadvantages include no widely-used and standardized saliva collection devices, difficulties in interpretation of saliva assay, and the lack of proficiency controls [12]. Currently, multiple categories of analytes can be measured in saliva, including enzymes, electrolytes, metabolites, and proteins [13]. Among them, certain analytes, such as cortisol and c-reactive protein, have already been used in dog studies for evaluation of stress and inflammatory lesions [14,15]. 

In view of the important functions of OT, the measurement of its level in biological samples has become of interest. To date, varieties of measurement methods have been developed in different categories including enzyme-linked immunosorbent assays (ELISA), radioimmunoassays (RIA), high performance liquid chromatography (HPLC), and liquid chromatography–mass spectrometry (LC-MS) [16,17,18,19,20,21]. However, indirect methods such as ELISA and RIA are criticized for their non-specific measurement mechanism and are sensitive to interference from other peptides, while methods using traditional HPLC without an MS detector face issues like low sensitivity and resolution. LC-MS, because of its high resolution and sensitivity, has been applied increasingly in nearly all areas of biological analysis. Even though LC-MS possesses advantages in many respects compared to other analytical platforms, the development of a reliable LC-MS method with both simplicity and sensitivities for OT measurement is challenging work. Researchers have already done several studies to develop LC-MS methods for OT measurement. However, those methods had certain limitations. For example, some studies had particular equipment requirements and relatively complicated sample preparation for blood OT measurement. One study used hydrophilic-lipophilic balance (HLB) solid-phase extraction (SPE) for sample preparation and a two-dimensional (2D)-LC-MS for OT detection [19]. Another study built a platform containing automated filtration/filter backflushing (AFFL), an on-line SPE, and a nanoLC-MS system [20]. Regarding the measurements of salivary OT, references using the LC-MS method are limited. One previously reported method used on-line in-tube solid phase microextraction (SPME) to couple with LC-MS for measuring salivary OT [21]. 

The purpose of this study was to develop and validate a simple LC-MS analytical method for the quantification of OT in dog saliva. After validation, the present LC-MS method was successfully applied to evaluate OT concentrations in dog saliva from multiple collections. 

## 2. Results and Discussion

### 2.1. LC-MS Conditions and Sample Preparation

**Figure 1:**
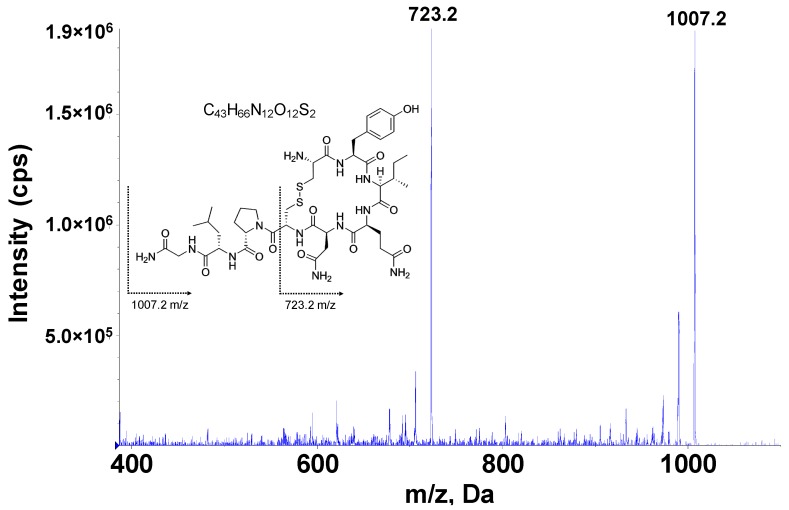
Mass spectra of oxytocin (OT) and its product ion. Spectra were acquired from the product ion scan. Collision energy ramped between 40–50 V.

In order to quantify OT concentrations in dog saliva, a reliable and robust assay was developed first. The LC-MS conditions and sample preparation procedure were carefully evaluated to obtain the optimal performance. The MS conditions for multiple reaction monitoring (MRM) were first optimized by direct infusion of OT in 50% aqueous acetonitrile (ACN) containing 0.1% (*v*/*v*) acetic acid (AA) into the system to select the ideal product ion and obtain the optimal signal intensity. Through the initial tuning experiment, the transition 1007.2 → 723.2 mass-to-charge ratio (*m*/*z*) was selected for OT quantification (Figure 1). This transition was also used by multiple previous studies [19,20,21]. At the same time, 1012.2 → 723.2 *m*/*z* was selected for monitoring OT-d_5_ signal. During the tuning process, the MS and MRM parameters were systematically optimized to enhance the response of OT in the system. After that, chromatographic conditions were evaluated for optimal performance. Formic acid (FA), AA, and trifluoroacetic acid (TFA) are commonly used as mobile phase additives for compounds that were detected in the positive ion mode. In the present study, OT spiked in 50% aqueous ACN with different levels of FA, AA, and TFA were directly infused into MS system to evaluate the responses of OT. Among the different conditions that were evaluated, the presence of 0.1% AA provided the highest signal intensity among the three additives and was then applied in the mobile phase preparation (Supplemental Figure S1). The ACE Excel C18 column was selected among several options for its assistance in resolution and signal intensity. Other chromatographic conditions like gradient and flow rate were evaluated for sharper peak shapes and higher signal intensities. Using the finalized chromatographic condition, the peak width at base was 4.2 s. 

**Figure 2:**
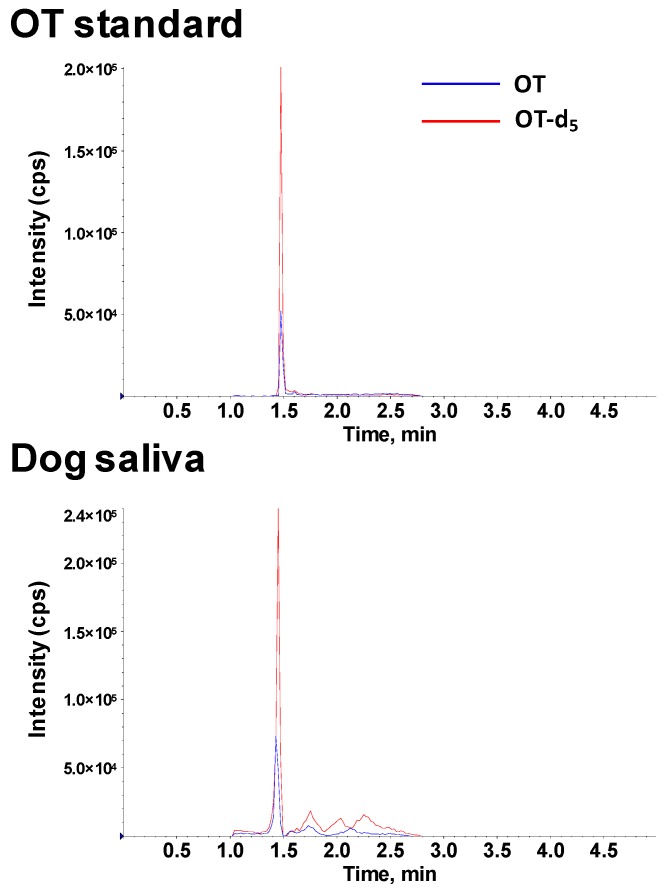
Representative chromatograms of standard solution and dog saliva sample. Blue line represent OT (1007.2 → 723.2 *m*/*z*), red line represent OT-d_5_ (1012.2 → 723.2 *m*/*z*). OT standard solution contained 500 pg/mL OT. A 300 µL of OT standard solution or dog saliva was extracted with 1.2 mL 80% aqueous ACN containing 1000 pg/mL OT-d_5_.

Sample extraction is also a critical step for salivary OT analysis. In the present study, a protein-precipitation-based saliva preparation method was developed. Protein precipitation is a common sample preparation process for measuring compounds in biological samples. A previous study suggested that OT did not precipitate with other bigger proteins or peptides during the protein precipitation process [19]. In addition, OT-d_5_ was added into the precipitation solution during the sample preparation process to facilitate the measurement of OT. After evaluations, 80% aqueous ACN was selected as a protein precipitation solution for its performance on recovery efficacy. After extraction, samples were completely dried and reconstituted. For reconstitution, 50% aqueous ACN was selected for the best signal intensity performance. After all optimization steps, the current sample preparation procedure and LC-MS settings worked well for OT detection. Representative chromatograms of OT standard solution and dog saliva sample are shown in Figure 2. 

### 2.2. Validation Results

#### 2.2.1. Linearity, Limit of Detection (LOD), and Limit of Quantification (LOQ)

The linearity of the methods was calculated using the internal standard (IS) calibration method and evaluated over a concentration range of 50–20,000 pg/mL in three validation runs. The calibration curves were constructed by plotting the OT versus OT-d_5_ area against the nominal concentrations of the calibration standards and were found to be linear with a mean regression coefficient of determination r^2^ ≥ 0.9996 (Supplemental Figure S2). To determine the LOD and LOQ, a sample (50 pg/mL OT in 50% aqueous ACN) was measured more than 6 times to calculate the LOD and LOQ. LOD was determined to be 9.0 pg/mL and LOQ was determined to be 36.6 pg/mL.

#### 2.2.2. Precision and Recovery

The intra and inter-day precision were determined using four samples spiked with different OT concentrations: LOQ (40 pg/mL), low (200 pg/mL), medium (400 pg/mL), and high (800 pg/mL). Intra-day precision was determined in one day (n ≥ 6), while inter-day precision was determined by runs on six consecutive days (n ≥ 12). The intra and inter-day precision were found to be ≤6.2% and ≤9.9%, respectively (Table 1). Recovery for OT was also assessed at the same four spiked concentration levels (40, 200, 400, and 800 pg/mL) and was calculated at 93.0, 92.7, 87.8, and 96.2% for each concentration level (Table 1). These data further confirmed that the current method was able to quantify OT with high accuracy.

#### 2.2.3. Matrix Effect

The matrix effect was determined by comparing the signals of OT spiked into the reconstitution of dog saliva extract with those of OT spiked in 50% aqueous ACN at the same concentration. The matrix effects were calculated to be 94.4% and 95.8% for OT at the two spiked concentration levels (200 and 800 pg/mL), which was acceptable (Table 2). 

#### 2.2.4. Stability

To evaluate the stability of the method, dog saliva samples spiked with OT (200 and 800 pg/mL) were kept at room temperature (RT) and 4 °C for 4 and 24 h. Those samples were also tested for stability after three cycles of freezing (−80 °C for 24 h) and thawing (25 °C for 15 min). Keeping saliva samples at RT for 24 h affected stabilities for both concentrations (Table 3). However, compared to long-term storage at RT, the stability was less affected if saliva samples were kept at RT for less than 4 h, or kept at 4 °C (Table 3). Additionally, freeze–thaw cycles had an effect on stability that was about 5% reduction (Table 3). As the step for extraction of saliva samples is straightforward and fast, there should be no major concern on the stability of the current method. Nevertheless, it is still recommended to minimize the freeze–thaw cycles and keep samples on ice during sample processing.

### 2.3. Comparison to the Existing LC-MS Methods

Several LC-MS methods for OT measurement were developed in previous studies. In one study, a straightforward LC-MS^n^ method was developed for measuring OT concentration in a dilute IV solution [22]. However, with an LOQ of 7.3 ng/mL, the method was not capable of quantifying OT in biological samples. Two other methods were developed for plasma OT quantitation using a LC-MS platform containing two LC systems. Both methods applied the SPE technique for sample preparation. One study reported an LOQ of 1.0 pg/mL for human plasma and 50.0 pg/mL for rat plasma [19]. No LOQ was reported from the second study. However, the linearity of the method was reported to be 5–2000 pg/mL [20]. Even though they demonstrated capabilities for the quantifying a low level of OT, those two methods involved special LC equipment requirements and multiple sample preparation processes, which were not suitable for laboratories with only common LC-MS systems. In another study, saliva was treated with procedures including evaporation and reconstitution and then introduced into an in-tube SPME device for extraction of OT before LC-MS analysis. The method was characterized to have an LOQ of 30 pg/mL [21]. However, without using an internal standard, this method had issues in identifying the OT peak in the chromatogram of actual saliva samples. Overall, compared to previously reported methods, the method that we reported here applied a simple protein precipitation-based procedure and demonstrated a comparable performance for OT quantitation.

### 2.4. Measurement of OT in Dog Saliva

**Figure 3:**
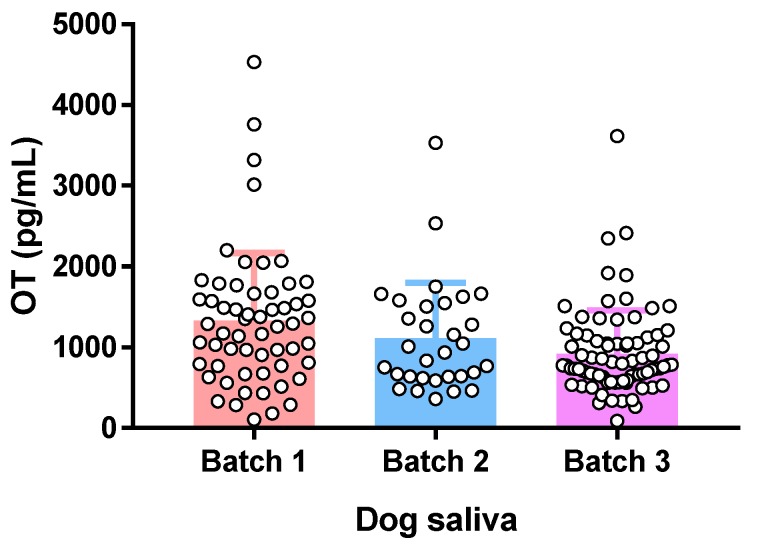
OT concentrations in dog saliva samples. Each batch contains 30–80 individual saliva samples. Data are represented as mean ± SD.

In order to prove its applicability, the validated method was further tested for OT measurement in dog saliva samples. The absorbent pad of a Versi·SAL® saliva collector was put in the cheek pocket of the dog for two minutes to collect saliva samples. Three batches of dog saliva samples were collected and analyzed. As shown in Figure 3, OT concentrations of individual dog samples were all above LOQ and quantified. These OT concentrations were within the same order of magnitude as those reported in a previous dog study [23]. This result indicates that the present method has sufficient sensitivity to determine OT concentrations in dog saliva samples. Previous studies found that variations in the sample collection procedure affected the quantitation of certain analytes in saliva [24,25]. In the present study, the influence of saliva collection procedure on OT concentration was not evaluated. Whether the collection procedure affects OT concentration in saliva or not may need investigation in the future. Additionally, in view of the successful measurement of OT in dog saliva using the present method, whether it can be applied for human saliva analysis requires further study.

## 3. Materials and Methods 

### 3.1. Reagent and Materials

OT acetate salt hydrate (≥97%) and stable isotope-labeled IS of OT (OT-d_5_ trifluoroacetate, ≥98 atom % D, ≥95% (CP)) were purchased from Sigma Aldrich (St. Louis, MO, USA). Optima^TM^ LC-MS grade water, ACN (≥99.9%), AA (≥99.7%), FA (≥99.0%), and TFA (≥99.5%) were acquired from Fisher Scientific (Pittsburgh, PA, USA). 

### 3.2. Saliva Collection and Preparation of Calibration Curve

Dog saliva samples were collected using Versi·SAL® kits from Oasis Diagnostics® Cooperation (Vancouver, WA, USA). Pooled dog saliva samples were used for method development and validation. Stock solutions of OT and OT-d_5_ were prepared with LC-MS grade water at a concentration of 2 and 1 mg/mL, respectively. OT calibration standards were prepared using 50% aqueous ACN from OT stock standard solution, with concentrations of 50, 100, 200, 500, 1000, 5000, 10,000, and 20,000 pg/mL.

### 3.3. Sample Preparation for LC-MS Analysis

A 300 µL of calibration standard or saliva sample was mixed with 1.2 mL 80% aqueous ACN containing IS (1 ng/mL OT-d_5_) in a 2 mL Eppendorf tube. The mixture was vortexed for 30 s and then centrifuged at 15,000× *g* under 4 °C for 10 min. After the centrifugation, the supernatant was transferred into another 2 mL Eppendorf tube and dried using a miVac sample concentrator (SP Scientific, Stone Ridge, NY, USA). After being completely dried, the sample was reconstituted with 50 µL 50% aqueous ACN. After another centrifugation at 15,000× *g* under 4 °C for 2 min, the supernatant was transferred to an HPLC vial for LC-MS analysis. 

### 3.4. LC-MS Analysis

A 10 μL of aliquot prepared from calibration standard or saliva sample was injected into a Shimadzu Nexera X2 ultra-high-performance liquid chromatography (UHPLC) (Shimadzu, Columbia, MD, USA) and separated on an ACE Excel C18 column (2 µm, 50 × 3 mm) (Advanced Chromatography Technologies Ltd, Aberdeen, Scotland) with a flow rate of 0.5 mL/min at 40 °C. Mobile phase A was water with 0.1% (*v*/*v*) AA and mobile phase B was ACN with 0.1% (*v*/*v*) AA. The mobile phase gradient ranged from water to 90% aqueous ACN over a 5 min run. It was started with 15% mobile phase B for 0.5 min, then increased to 60% within 0.5 min, increased to 70% within 1 min, then kept constant 90% for 1.5 min, reduced to 15% at 4 min, and kept constant until the end of the run at 5 min (Supplemental Table S1). Autosampler temperature was kept at 10 °C. The LC eluent was introduced into an AB Sciex 6500+ quadrupole ion trap mass spectrometer (QTRAPMS) (AB Sciex, Framingham, MA, USA) for MRM using positive ion mode with electrospray ionization (ESI). Final MS and MRM parameters were as follows: Curtain gas (25psi), collison gas (high), IonSpray voltage (3500 V), source temperature (400 °C), ion source gas 1 (50psi), ion source gas 2 (50psi), declustering potential (60 V), entrance potential (10 V), exit potential (20 V), collision energy (42 V), and dwell time (200 ms). Quantification was performed by MRM of the protonated precursor molecular ions [M + H]^+^ and the related product ions. The resolutions of quadrupole Q1 mass (precursor ion) and Q3 mass (product ion) were set at high. For MRM scan of OT, ion pairs 1007.2 → 723.2 *m*/*z* were monitored for quantitation, while ion pairs 1007.2 → 621.0 *m*/*z* were monitored for confirmation. The MRM scan for OT-d_5_ monitored ion pairs 1012.2 → 723.2 *m*/*z*. Chromatograms and mass spectral data were acquired and processed using Analyst® 1.6.3 software (AB Sciex, Framingham, MA, USA). 

### 3.5. Method Validation

The developed method was validated for the calibration curve performance, precision, matrix effects, and stability according to the Nestlé guidelines and referring to bioanalytical method validation guidelines of the US Food and Drug Administration (FDA).

#### 3.5.1. Linearity, LOD, and LOQ

The calibration curves were determined by using the ratio of the peak area of OT to the peak area of OT-d_5_ versus the concentrations of OT spiked in 50% aqueous ACN by a linear least squares regression model. The calibration was prepared at eight levels (50, 100, 200, 500, 1000, 5000, 10000, and 20,000 pg/mL). Linearity was evaluated by monitoring coefficient of determination (r^2^) of the calibration curves. The performance of the assay was also characterized by determining the LOD and the LOQ. Samples with a low amount of analyte (50 pg/mL OT in 50% aqueous ACN) were measured more than 6 times to calculate the LOD that is 3 times that of the standard deviation of measured concentrations and LOQ that is 10 times that of the standard deviation of measured concentrations. 

#### 3.5.2. Precision and Recovery

Precision was evaluated by measuring the coefficient of variation (CV) of the measurements. Recovery was determined by comparing the measured concentration of OT to the theoretical concentration of OT spiked into the pooled saliva. Both precision and recovery were investigated on four spiked levels including LOQ (40 pg/mL), low (200 pg/mL), medium (400 pg/mL), and high (800 pg/mL). The measurements were performed in one day (intraday, n ≥ 6) and duplicates in more than 6 consecutive days (interday, n ≥ 12). 

#### 3.5.3. Matrix Effect

The influence of matrix effect on the OT measurement was evaluated by comparing the measured concentration of OT at a low (200 pg/mL) and high (800 pg/mL) levels spiked in triplicate into extract reconstitution of the pooled dog saliva to the measured concentration of OT spiked in triplicate into the 50% aqueous ACN. 

#### 3.5.4. Stability

The stability of OT in saliva was evaluated by analyzing low (200 pg/mL) and high (800 pg/mL) spiked samples after stored at room temperature and 4 °C for 4 and 24 h. Freeze–thaw stability was tested after three cycles of freezing (−80 °C for 24 h) and thawing (25 °C for 15 min). 

### 3.6. Application of Method to Dog Saliva Samples 

To demonstrate the applicability, the validated method was tested for measuring OT concentrations in saliva samples collected from individual dogs with different ages and breeds. Versi·SAL® kits were used to collect saliva from dogs for 2 min. After collection, saliva samples were transferred to individual Eppendorf tubes and stored at −80 °C before analysis. 

## 4. Conclusions

A simple and sensitive LC-MS method was developed and validated for the determination of OT concentration in dog saliva. The method was characterized to be with high sensitivity (LOQ at 36.6 pg/mL) and high reliability over the concentration range of 50–20,000 pg/mL. To the best of our knowledge, this is the first study to report that a simple protein precipitation based LC-MS method was validated to be with low LOQ and high accuracy for OT measurement in dog saliva. What is more, the method was successfully applied to measure OT concentrations in hundreds of dog saliva samples. The accurate and sensitive method reported here could be a promising tool to facilitate studies aiming to use OT as a biomarker for human–animal interaction studies. 

## Figures and Tables

**Table 1:** Intra- and interassay CV ^1^, and recovery of the present method for salivary OT.

Sample	Spiked (pg/mL)	Recovery	Intraassay CV	Interassay CV
LOQ	40	93.0% (37.2 ± 4.2)	6.2% (37.4 ± 2.3)	9.9% (36.9 ± 3.8)
Low	200	92.7% (185.3 ± 35.9)	5.4% (187.5 ± 10.1)	5.5% (182.0 ± 10.6)
Medium	400	87.8% (351.1 ± 38.2)	5.7% (360.4 ± 20.5)	4.7% (348.8 ± 19.8)
High	800	96.2% (769.7 ± 78.5)	1.6% (781.5 ± 12.9)	9.6% (763.1 ± 83.0)

^1^ CV, coefficient of variation;

**Table 2:** Matrix effects of OT spiked in dog saliva.

Sample	Spiked (pg/mL)	Matrix Effects (%)	CV ^1^ (%)
Low	200	94.4 ± 4.3	4.6
High	800	95.8 ± 14.3	14.9

^1^ CV, coefficient of variation.

**Table 3:** Stability of OT spiked in dog saliva.

Sample	Spiked (pg/mL)	3 Cycles Freeze/Thaw (−80 °C/RT ^1^)		RT, 4 h		RT, 24 h		4 °C, 4 h		4 °C, 24 h	
		Mean (%)	CV ^2^ (%)	Mean (%)	CV (%)	Mean (%)	CV (%)	Mean (%)	CV (%)	Mean (%)	CV (%)
Low	200	94.9 (171.1 ± 19.0)	11.1	99.3 (179.0 ± 22.9)	12.8	87.6 (157.8 ± 21.4)	13.6	97.8 (176.2 ± 20.8)	11.8	97.6 (175.9 ±2.5)	1.4
High	800	96.5 (689.9 ± 17.2)	2.8	99.5 (708.9 ± 19.9)	3.2	92.6 (665.2 ± 34.2)	5.8	98.9 (705.4 ±64.9)	10.4	98.6 (702.9 ±54.7)	8.8

^1^ RT, room temperature; ^2^ CV, coefficient of variation.

## Supplementary Materials

The following are available online at https://www.mdpi.com/1420-3049/24/17/3079/s1. Figure S1: OT signals in MS using different mobile phase additives, Figure S2: Calibration standard curve of OT, Table S1: LC parameters.
