# Supplementary material for: Development and Validation of a Simple LC-MS Method for the Quantification of Oxytocin in Dog Saliva
Source: Molecules. 2019 Aug 24;24(17):3079. doi: 10.3390/molecules24173079 (PMC6749683; doi:10.3390/molecules24173079)
Supplement: Supplementary file 1 [file molecules-24-03079-s001.pdf]

Development and validation of a simple LC-MS method for the quantification of oxytocin in dog saliva

Lei Wang*, Dakota W. Marti, Rachel E. Anderson

Nestlé Purina Research, Nestlé Purina PetCare, St. Joseph, MO 64503, USA.

*To whom correspondence should be addressed**:**

Lei Wang, Nestlé Purina Research, Nestlé Purina PetCare, 3916 Pettis Road, St. Joseph, MO 64503, USA. Tel.: 816-387-4127; Email: Lei.Wang@rd.nestle.com.

Supplemental Table S1: LC parameters

LC Parameters:		
Mobile Phase:	A: 0.1% Acetic acid in water	
	B: 0.1% Acetic acid in ACN	
Gradient:	Time (min)	Mobile Phase B (%)
	0.01	15
	0.50	15
	1.00	60
	2.00	70
	2.01	90
	3.50	90
	4.00	15
	5.10	15
Flow rate:	0.5 mL/min	
Sample injection volume:	10 µL	
Column temperature:	40°C	

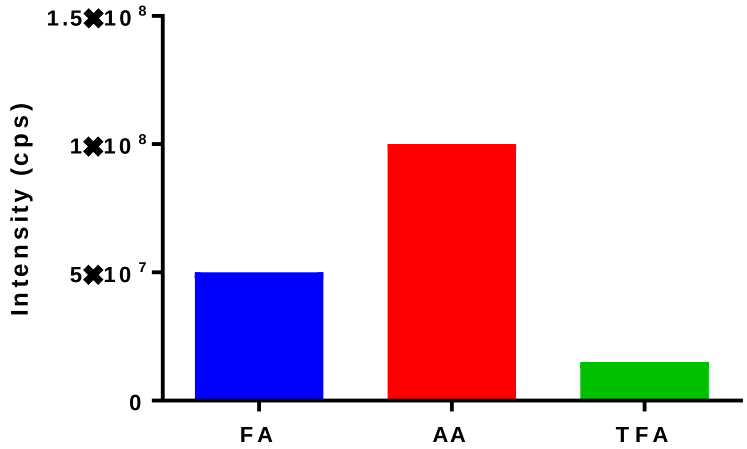

Supplemental Figure S1*:* OT signals in MS using different mobile phase additives. OT (100 ng/mL) was prepared in 50% aqueous ACN containing 0.1% formic acid (FA), 50% aqueous ACN containing 0.1% acetic acid (AA), or 50% aqueous ACN containing 0.1% trifluoroacetic acid (TFA). Samples were directly infused into MS system to acquire OT signal.

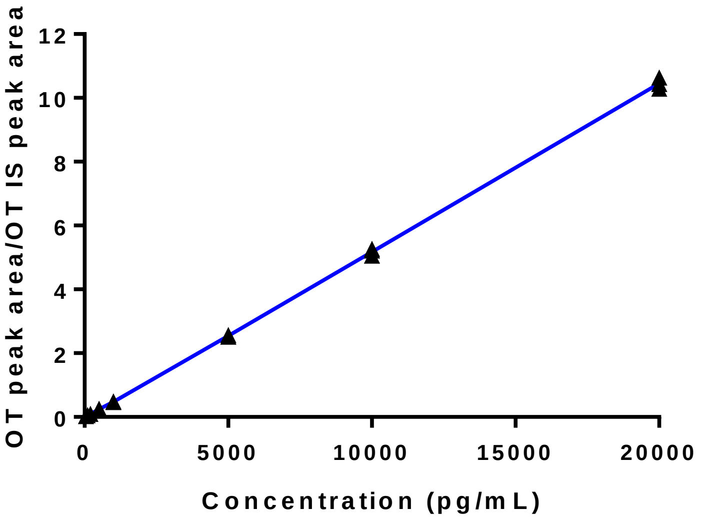

Supplemental Figure S2: Calibration standard curve of OT. Standard peak area ratio (peak area analyte/peak area internal standard) was used for calibration calculation. The standard concentrations included 50, 100, 200, 500, 1000, 5000, 10000, and 20000 pg/mL. All standards were prepared in triplicate. Linear equation: y=0.51768x-0.00051. Coefficient of determination: $r^2 = 0.9996$.
